# Implementation of Online Research Training and Mentorship for Sub-Saharan African Family Physicians

**DOI:** 10.5334/aogh.3171

**Published:** 2021-02-03

**Authors:** Chelsea M. McGuire, Bolatito B. Fatusin, Hithaishini Kodicherla, Kenneth Yakubu, Pius Ameh, Alexandra van Waes, Ethan Rhoad, Brian W. Jack, Nancy A. Scott

**Affiliations:** 1 Department of Family Medicine, Boston University, Boston, USA; 2 Family Medicine Specialty Training Program, Lesotho-Boston Health Alliance, Leribe, Lesotho; 3 Institute for Health System Innovation and Policy, Boston University, Boston, USA; 4 Family Medicine Department, Federal Medical Centre, Gusau, Nigeria; 5 Boston University School of Public Health, Boston University, Boston, USA; 6 Department of Family Medicine, University of Jos, Jos, Nigeria; 7 Faculty of Medicine, University of New South Wales, Sydney, Australia; 8 The George Institute for Global Health, Sydney, Australia; 9 Department of Family Medicine, Federal Medical Centre, Keffi, Nigeria; 10 Boston University Department of Health Sciences, Boston University, Boston, USA; 11 Boston University School of Public Health, Department of Global Health, Boston University, Boston, MA, USA

## Abstract

**Background:**

To improve the delivery and reach of primary health care, a robust scientific foundation driven by research is needed. However, few family physicians conduct research, especially in sub-Saharan Africa. Early-career and trainee family physicians are a key part of the primary care research pipeline and have an expressed need for research training and mentorship.

**Objective:**

AfriWon Research Collaborative (ARC) was an online research training and mentorship pilot program whose objective was to increase research activity among participants from AfriWon Renaissance, the family physician young doctors’ movement of sub-Saharan Africa.

**Methods:**

ARC utilized a 10-module online curriculum, supported by peer and faculty e-mentorship, to guide participants through writing a research protocol. The feasibility, acceptability, and scalability of this program was evaluated via a mixed-methods RE-AIM-guided process evaluation using descriptive statistics and inductive/deductive thematic analysis.

**Findings:**

The pilot reached participants from Botswana, Democratic Republic of the Congo, Ghana, Nigeria and Sierra Leone and was adopted by mentors from 11 countries across three continents. Four of the 10 pilot participants completed a full research protocol by the end of the six-month core program. Seven out of the 10 participants, and nine out of the 15 mentors, planned to continue their mentorship relationships beyond the core program. The program helped instill a positive research culture in active participants. Some participants’ and mentors’ engagement with the ARC program was limited by confusion over mentorship structure and role, poor network connectivity, and personal life challenges.

**Conclusions:**

Online research training and mentorship for trainee and early-career family physicians in sub-Saharan Africa is feasible and acceptable to participants and mentors. Similar programs must pay careful attention to mentorship training and provide a flexible yet clearly organized structure for mentee-mentor engagement. Additional work is needed to determine optimal implementation strategies and ability to scale.

## INTRODUCTION

For primary health care to achieve its promise of improving health, reducing cost, and decreasing inequity [1], primary care research capacity must be strengthened globally [2]. Primary care researchers provide needed information about planning, monitoring, and evaluating a country’s health system [3]. Clinician-researchers in primary care, especially generalist Family Physicians (FPs), conduct research that is highly relevant to people, families, and communities [3,4].

Around the world, primary health care research is limited by a lack of skilled researchers, training opportunities, and resources, with these challenges being further amplified in sub-Saharan Africa [4,5]. Recommendations for increasing FP research activity include increasing enrollment in doctoral research programs, ensuring all family medicine departments set a research agenda, and building a stronger research culture by integrating service, learning, and research [3,4]. In sub-Saharan Africa, FP postgraduate training programs require a research project or thesis [6]. Exposure to research during training could build a foundation for implementing the recommendations above. However, many sub-Saharan African FP training programs have limited available local mentorship and research expertise [4,7]. Without proper training, mentorship, and sufficient allocated time, the research thesis can become a burdensome obligation that could contribute to already elevated levels of burnout and discourage further research activity [4,8,9].

Peer research mentorship has successfully filled gaps in mentorship within academic institutions where the small number of research faculty are over-burdened [10]. Health research training through distance learning is increasingly used as a way to expand access in low resource settings [11]. Research mentorship is also moving online, since e-mentorship can help overcome distance and time constraints [12]. AfriWon Renaissance, or AfriWon, is the young doctors movement within the African region of the World Organization of Family Doctors (WONCA) and is comprised of current postgraduate FP trainees and early-career FPs within five years of graduation [13]. Collaborative research and opportunities for mentorship are among the top reasons young FPs join groups like AfriWon [14].

The feasibility, acceptability, and scalability of an online research training program that provides peer and faculty e-mentorship to early-career FPs in sub-Saharan Africa, where internet connectivity and research mentorship experience tend to be limited, is unknown. In response to this need, members of this study partnered with AfriWon to develop and pilot test the AfriWon Research Collaborative (ARC) research training and mentorship program. This is the first of a series of papers on the ARC program. This paper describes the program components and reports on key implementation indicators.

## METHODS

### PROGRAM DESCRIPTION

**Figure 1:**
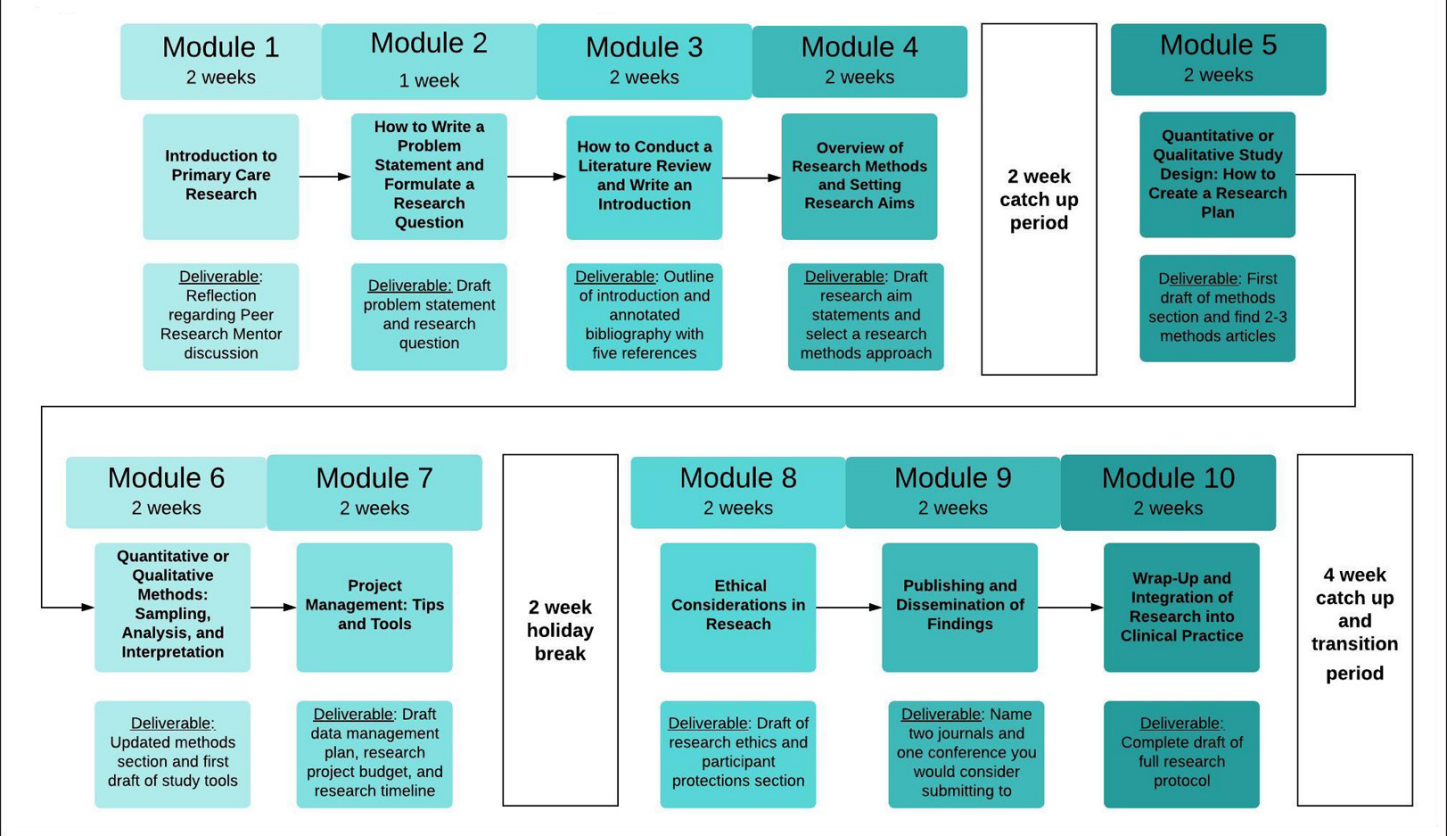
AfriWon Research Collaborative Pilot Program Curriculum and Timeline.

**Figure 2:**
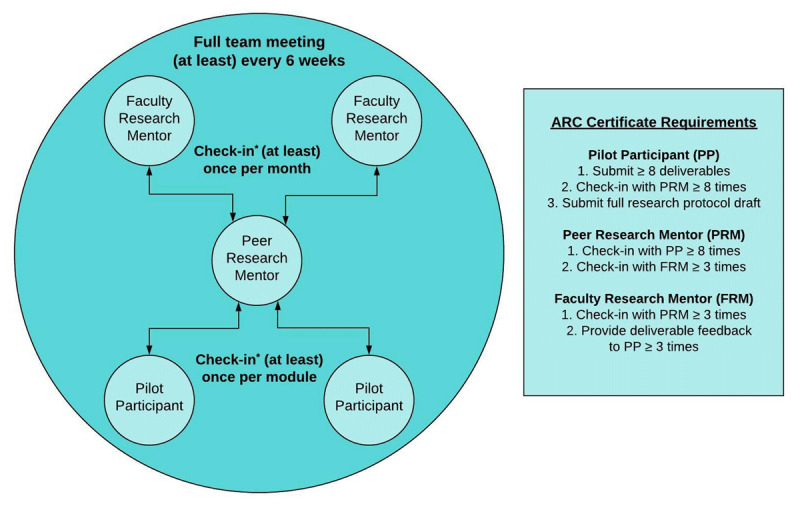
AfriWon Research Collaborative Mentorship Structure, Communication Guidelines, and Certificate Requirements. Abbreviations: PP, Pilot Participant; PRM, Peer Research Mentor; FRM, Faculty Research Mentor. * Mentorship outreach by mentor or mentee that takes place via email, text message, voice or video call is considered a check-in.

The ARC pilot program was designed to increase participants’ research activity through an online curriculum (***Figure 1***), supported by an e-mentorship team (***Figure 2***). The six-month pilot ran from September 2019 to March 2020 and was limited to 10 participants. ARC’s program development was guided by two logic models that mapped the development of needed inputs, activities, outputs, and anticipated outcomes (***Supporting Figures S1.1, S1.2***). Participants and mentors received a certificate of completion for meeting a priori criteria for engagement in the program (***Figure 2***).

#### Curriculum

The ten-module ARC research-training curriculum was designed to guide participants step-by-step through writing a primary health care research proposal (***Figure 1***). Each module contained one or more recorded screencast lectures, supplemental reading materials, and short assignments or “deliverables” and was accessed via Google Classroom (Google LLC., Mountain View, California, USA). All participants and peer mentors were provided with an e-textbook *How to do Primary Care Research*, which also informed the curriculum design [15]. Lectures averaged 30 minutes each and were delivered by WONCA research working party members, including four from sub-Saharan Africa, and from Boston University faculty.

#### Mentorship

The suggested ARC mentorship structure was tiered in order to support mentorship skill development. Peer research mentors provided direct mentorship to pilot participants. Faculty research mentors provided direct mentorship to the peer mentors and indirectly to the participants (see mentorship criteria in ***Box 1***). Small groups of participants, peer mentors, and faculty mentors formed mentorship teams and were given guidelines for communication frequency (***Figure 2***).

**Box 1 Inclusion, Selection, and Exclusion Criteria for AfriWon Research Collaborative Pilot Program Participants and Mentors**

**Inclusion Criteria for Pilot Participants:**

1. Be a trainee or early-career family physician who is within five years of qualification
2. Live in sub-Saharan Africa
3. Have no first-author, peer-reviewed research publications

**Selection Criteria for Pilot Participants:**

1. Identification of a clear learning/competency need in research
2. Demonstrated passion to learn
3. Expressed need for mentorship
4. Desire for a regular research practice beyond family medicine training

**Inclusion Criteria for Peer Research Mentors:**

1. Be a trainee or early-career family physician who is within five years of qualification
2. Have at least one first-author, peer-reviewed research publication

**Inclusion Criteria for Faculty Research Mentors:**

1. Be a family physician who is at least five years post-qualification
2. Have at least five first-author, peer-reviewed publications

**Exclusion Criteria for All Groups (Self-determined):**

1. Lack of proficiency in reading, writing and/or speaking the English language; or
2. Insufficient access to the internet to participate in training and/or mentorship activities

WhatsApp (Facebook, Inc., Menlo Park, California, USA) groups for each of the five teams were created for this study. Additional WhatsApp groups were created for participants and also for peer mentors to provide the opportunity to support each other horizontally across the program. Peer mentors, who were expected to check-in with their participants at least once per module, were given a professional Zoom account to host mentorship meetings (Zoom Video Communications, San Jose, California, USA). During the program orientation, both peer and faculty mentors were trained in the “spirit” of motivational interviewing [16] as the foundation of ARC mentorship approach. In addition to providing research guidance, an explicit objective of ARC mentors was to build mentee’s resiliency and combat burnout. Participants were encouraged to communicate with local research supervisors and other local collaborators where applicable.

#### Organization and management

The ARC pilot program was developed, implemented, and evaluated by the ARC working group, which met bi-weekly and included co-authors CMM, BBF, HK, KY, PA, LVW, and ER. One working group member served as a liaison to each ARC mentorship team to support implementation and evaluation at the team level. An ARC advisory group, composed of stakeholders from WONCA, AfriWon, FP training program faculty within sub-Saharan Africa, and Boston University advisors, including co-authors NAS and BWJ, met quarterly to provide expert guidance to the working group.

### RECRUITMENT, SELECTION, AND MATCHING

Participants were recruited from the membership of AfriWon Renaissance and from the FP training program in Botswana. ***Box 1*** details the program inclusion and exclusion criteria, as well as selection criteria for participants. Recruitment for participants and peer mentors occurred primarily via WhatsApp and Facebook (Facebook, Inc., Menlo Park, California, USA). Additionally, recruitment of faculty and participants occurred in-person at the 2019 WONCA Africa conference and through email. Interested individuals first submitted a brief electronic form that evaluated inclusion criteria. Those who met inclusion criteria were sent an electronic application. The working group conducted a blinded rating of completed applications to select the participants. All peer mentors who completed the application were accepted. Faculty mentors who completed the application were accepted on a rolling basis until all positions were filled.

To match the mentorship teams, participants were provided with brief profiles of prospective peer and faculty mentors and asked to rank their choices. The working group created the mentorship teams based on a combination of participant preferences, research interests, research methods expertise, and location. Each team had at least one mentor from within sub-Saharan Africa to ensure participants were exposed to successful family medicine researchers from contexts similar to their own.

### PROCESS EVALUATION

The ARC pilot was evaluated by a mixed-methods, prospective process evaluation that focused on monitoring and evaluation to allow for real-time adaptation. Individual, team, and program level implementation indicators were assessed to understand acceptability, feasibility, and scalability of the program.

#### Evaluation framework

The RE-AIM implementation framework was selected to guide the evaluation because it offers a systematic way to assess key design and implementation processes [17]. This process evaluation paper focuses on three of the RE-AIM domains: reach (R), adoption (A), and implementation (I). ***Box 2*** depicts how these domains have been adapted to the ARC pilot and the indicators used to measure each.

**Box 2 Adapted Relevant RE-AIM Framework [17] Definitions and Indicators within ARC Pilot Program and Evaluation**

**Table:** 

Domain	Adapted definition and indicators for ARC pilot
Reach	The number, demographic characteristics, and geographic spread of the PPs within AfriWon Renaissance; reasons for participating; linked to target logic model construct “Working group recruits PPs.”
Adoption	The number, demographic characteristics, and geographic spread of mentors within regions of WONCA; reasons for adoption; linked to the non-target logic model construct, “Working group recruits FRMs and PRMs”
Implementation	Includes: 1. Individual level implementation (measured by PP deliverable completion), 2. Team level implementation (measured by team communication frequency and mentorship structure), and 3. Program level implementation (measured by a series of indicators capturing adaptations made and resources needed, including time, money and skills); linked to nearly all inputs, activities, outputs, and short-term outcomes in the target logic model

Abbreviations: PP, Pilot Participant; WONCA, World Organization of Family Doctors; FRM, Faculty Research Mentor; PRM, Peer Research Mentor.

#### Data sources, sampling and management

Data on participant deliverable submissions and team mentorship communication was collected at the end of each module. For team communication, the number of check-ins between each team member (***Figure 2***) was captured. Midpoint and final anonymous online surveys invited feedback from all participants and mentors. Program logs tracked recruitment, onboarding, program activities, adaptations, needed resources (financial, human, and time), and program observations by the working group. Data were extracted from program records and directly collected from participants, peer mentors, and faculty mentors. A table that outlines each data type, variables, and management strategy is provided as ***Supporting Table S2***.

#### Quantitative data analysis

Descriptive statistics of demographic data, including medians, frequencies, and ranges were calculated using RStudio (Version 1.2.1335) [18]. Missing values were omitted from calculations. To approximate participant and mentor representativeness, their geographic locations were compared to the regions represented within AfriWon and WONCA organizations, respectively. Deliverable completion and mentorship check-ins were analyzed descriptively at the participant, team, and program levels and compared to the requirements to receive an ARC program completion certificate.

#### Qualitative data analysis

All qualitative data collected via open-ended survey responses, as summary documents of program logs, as well as from meeting minutes and recordings from feedback group discussions were uploaded into NVivo (Version 12.6.0) for analysis [19]. Data were analyzed by thematic analysis using a mixed inductive-deductive approach [20]. All data were read and checked for errors by the coding team, CM, LVW, BF and HK, during the familiarization phase. An *a priori* codebook was developed using program implementation indicators and relevant RE-AIM domains [17]. All coders conducted independent open-coding to refine codebook definitions and identify new codes; the codebook was finalized with agreement by all. All qualitative data were then independently coded by two coders, reviewed by team members to identify common themes, and findings were mapped to the RE-AIM framework. These themes were then reviewed by the full working group to finalize the core findings. Memoing was used by coders throughout the qualitative analysis process [21]. This allowed for reflexivity regarding how the evaluators’ role in the program might influence its evaluation [22]. Triangulation of qualitative and quantitative data within and across each RE-AIM domain was used to increase validity of findings [23]. Additionally, member checking provided all participants and mentors the opportunity to comment on the findings [24].

### ETHICAL CONSIDERATIONS

Ethical approval was obtained from the Boston University Medical Campus Institutional Review Board in the USA (Protocol H-38521) and Federal Medical Center Keffi Health Research Ethics Committee in Nigeria (Reference FMC/KF/HREC/299/19). ARC orientation sessions included a verbal description of evaluation procedures, risks, and benefits. After orientation, all participants and mentors signed an online consent form prior to initiating the program.

## RESULTS

Quantitative followed by qualitative findings within each domain are presented here. Implementation domain results are reported at the individual, team, and program levels. Qualitative excerpts are shown in italics; those with quotation marks are direct quotes. Quotes are followed by the either the source of the aggregate data, or the code of the respondent, with pilot participants coded by “PP;” peer research mentors by “PRM;” and faculty research mentors by “FRM;” followed by a letter indicating the team, and a number indicating the unique individual.

### REACH

Twenty-one prospective participants were recruited, among whom eighteen met inclusion criteria and were sent applications. Twelve completed the application process and ten participants were selected. Nine of the ten participants were in-training and half were female (***Table 1***). The baseline self-reported burnout level varied, with one participant reporting “no burnout,” six experiencing occasional stress and/or low energy and three reporting they are “definitely burning out.” The ARC pilot reached participants from five countries across Western, Central, and Southern Africa, representing three of the four regions that make up AfriWon Renaissance.

**Table 1:** Demographic and Professional Characteristics of ARC Pilot Participants, Peer Mentors, and Faculty Mentors.


	PARTICIPANTS (n = 10^a^)	PEER MENTORS (n = 5)	FACULTY MENTORS (n = 10)

Age, median years (range)	37 (31–54)	39 (29–47)	50.5 (44–69)

Gender, n Female Male	5 5	2 3	— 10

Country practicing in, n Botswana Croatia Democratic Republic of Congo Denmark Ghana Liberia Netherlands Nigeria Sierra Leone South Africa Turkey Ukraine United States of America	3 — 2 — 3 — — 1 1 — — — —	— 1 — — — — — 1 — — 1 1 1	1 — 1 1 — 1 1 2 — 2 — — 1

First-author publications, n 0 1–5 6–10 ----------------- 11–30 31–50 >51	10 — — ———————— — — —	— 4 — ———————— — 1 —	— 2 4 ———————— 2 1 1

Years since finished training, n 0 1–5 6–10 11–15 16–20 21–25 >26	9 1 — — — — —	— 4 1 — — — —	— — 2 4 1 2 2


Abbreviations: ARC, AfriWon Research Collaborative; n, number. ^a^ Participants n = 10, except missing data as follows: Age n = 1.

The desire to increase one’s research capacity and access research mentorship were among the most commonly reported reasons participants applied to ARC:

*“I am interested in joining the ARC pilot program because I have attempted creating a proposal for my Fellowship thesis for quite some time without success. (…) I did not have the requisite skills nor understanding to do so. Close and structured mentorship in our setting is not readily available*.” (PPL5)

The baseline prior research experience varied widely among participants. Some reported: *“I have no experience with research*.” (PPH3) One had a research master’s degree, but no publications. Others cited exposure only in medical school: *“The only research project to my credit today is my thesis at the end of [my] medical study*.” (PPU1)

### ADOPTION

Eleven individuals were interested in becoming peer mentors; five completed the full application and onboarding process and were accepted. The peer mentors were a median of 4 years out of training (range 4 to 10) and had a median of 3 prior first-author, peer-reviewed publications (range 1 to 34). Thirteen individuals were interested in becoming faculty mentors; 11 completed the full application and 10 were accepted. The faculty mentors were a median of 13.5 years out of training (range 6 to 32) and had a median of 8.5 prior first-author, peer-reviewed publications (range 2 to 47). The 15 peer and faculty mentors came from eleven different countries, representing adoption of the ARC intervention by FP mentors from four of the seven regional groups within WONCA (***Table 1***) [25].

Both peer and faculty mentors reported joining ARC to support FP research capacity in Africa: *“I would like to join the ARC program to promote capacity-building for research in Africa – to support more research conducted and published by Africans in Africa*.” (FRMH1) Peer mentors reported wanting to collaborate globally on research, improve mentorship skills, and to support other early-career FPs and general practitioners (GPs):

*“As an early career GP researcher, I’m aware of the many barriers that exist that make it difficult for GPs to start doing research (…) I want to bring young researchers closer together and share the knowledge and experience that I have*.” (PRMH1)

Faculty mentors noted wanting to “give back” through mentorship. Two faculty mentors specifically highlighted an interest in distance mentorship: *“I feel I can help other students around the world with research activities since I have done this before remotely*” (FRML1); *“I’m interested in joining the ARC program (…) to gain experience in distance mentorship*.” (FRMX1)

### IMPLEMENTATION

#### Individual level

Four out of 10 pilot participants submitted the final deliverable, a full research protocol draft. One participant got as far as the ninth deliverable, selection of a target conference and journals. In all, eight participants submitted at least one deliverable; two submitted no deliverables. Research topics explored in these deliverables ranged from patient-centered research on hypertension and postpartum depression to health systems research on the use of electronic medical records. Participants reported a wide range of time spent on ARC activities, most falling between three to six hours per week.

Engagement in ARC increased participants confidence, competence, and interest in primary health care research: *“Participant 1 has improved in confidence to do research (…) She has also shown improvement in competence and can show clear evidence of understanding and applying the modules*” (Anonymous FRM, Midway Evaluation); *“The program did not only teach me how to do primary care research but it ignited my passion for it and helped me realize the need for it in my setting*.” (Anonymous PP, Final Evaluation) The modules and e-book aided in participant’s learning: *“I [went] back to the chapters that were not even part of the modules (…) so that my understanding of the modules could be broadened.”* (PPH8)

However, other participants’ engagement was limited by personal challenges, network connection, language, and time: *[The participant] was unable to complete the program after his bereavement* (Mentorship Summary 1); “*he told me that he had no connection (…) and then after that [he] is still quiet*.” (PRML1); *“I guess that language is quite a handicap for the mentee.”* (Anonymous FRM, Midway Evaluation); *“It has been a little [more] time consuming than expected. It was initially very difficult to create a balance between my routine residency work and the program.”* (Anonymous PP, Midway Evaluation)

#### Team level

**Figure 3:**
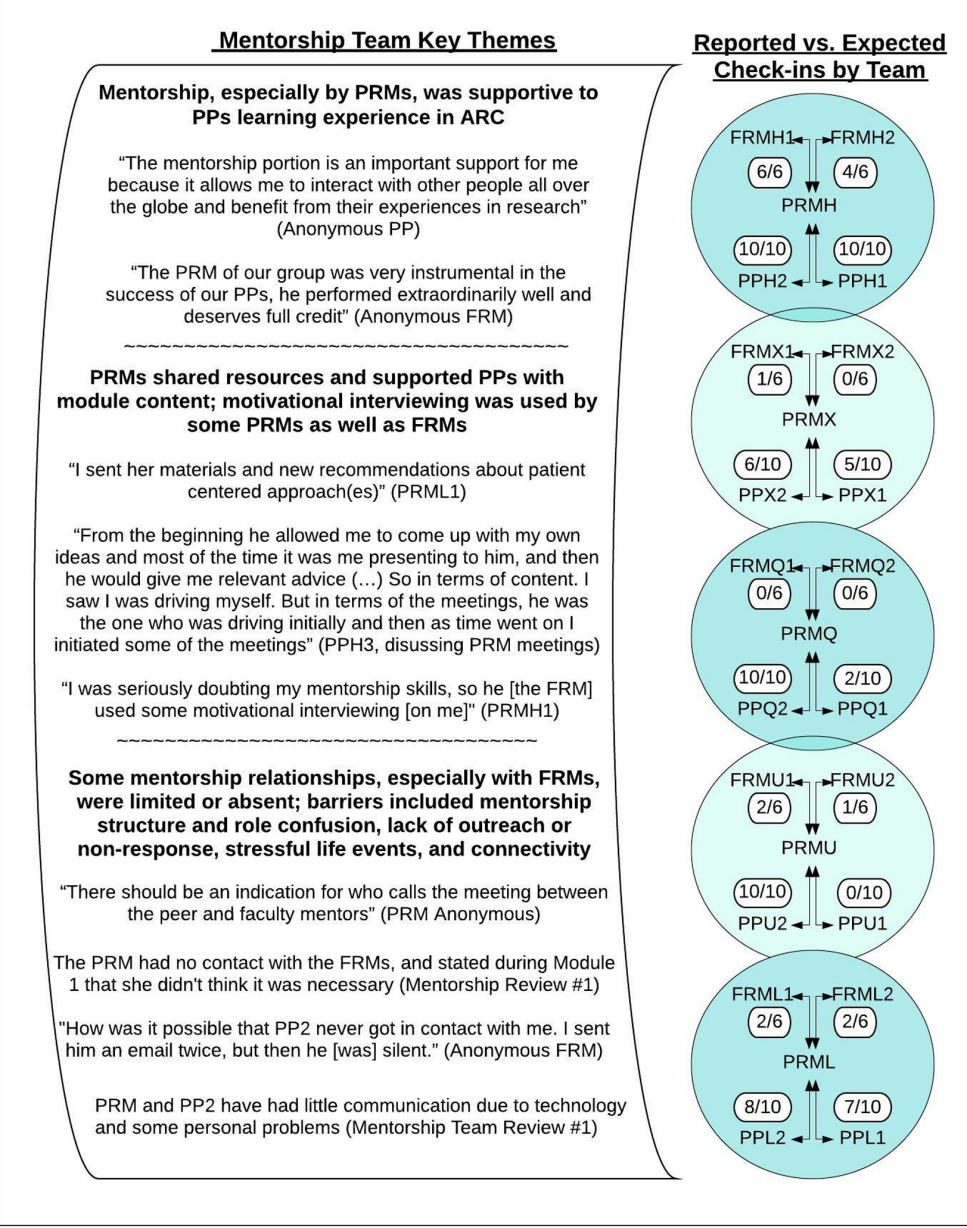
ARC Mentorship Team Qualitative Findings and Reported Mentorship Check-ins. Abbreviations: ARC, AfriWon Research Collaborative; FRM, Faculty Research Mentor; PP, Pilot Participant; PRM, Peer Research Mentor; Note: Quotations marks are indication of direct quote from participant or mentor.

Mentorship implementation by team was mixed. ***Figure 3*** shows reported check-ins on each team. One team had high levels of communication between all members of the mentorship team. Other teams had varying levels of communication between participants and peer mentors, which typically mirrored participants’ deliverable completion rates. Significantly less than expected communication between peer mentors and faculty mentors was observed.

The team with highest mentorship communication rates also reported implementing the suggested tiered mentorship structure (***Figure 2***) and three full team meetings. Another team implemented a tiered structure initially, but when the peer mentor had to stop at Module 8 for personal reasons, one of the faculty mentors became the primary mentor to the two participants. A third team reported using a more direct 1:1 mentoring relationship between each mentor and the participant. Two teams had little or no faculty mentor involvement in the mentorship structure.

As the key themes shown in ***Figure 3*** demonstrate, the mentorship relationships that formed during ARC were supportive to both participants and peer mentors alike. One peer mentor reported supporting his participant through a loss: *“It was a down moment for everyone in the hospital. It puts him off psychologically for some time. Eventually with motivational interviewing, he was able to pick it up*.” (PRMU1) While another peer mentor reported being guided in his mentorship approach by his faculty mentor:

*“[The faculty mentor] was able to answer any question I had. Like if you have two different participants, and one is behind, do you give more effort to the one who is behind or to both? I had a lot of questions like that (…) he gave me some really good advice*.” (PRMH1)

The lack of involvement of the faculty mentors on some of the mentorship teams was noted by participants and faculty mentors alike: *“Interaction with faculty mentors was too limited, positive/negative feedback would be appreciated with each deliverable*.” (Anonymous PP, Final Evaluation)*; “I commented on a few proposals of my mentee and phoned with him and the mentor in [country] and with the coordinator. But I feel very insufficient over my contribution.”* (Anonymous FRM, Midway Feedback)

#### Program level

Across the entire program, participants completed an average of 5.5 of 10 curriculum deliverables and checked in with their peer mentors an average of 5.8 times over the 10 module time periods. Overall, four out of 10 participants, three out of five peer mentors, and three of 10 faculty mentors met the completion certificate requirements over the six months. Seven participants, four peer mentors, and eight faculty mentors expressed interest in continuing their mentorship relationships beyond the core program.

There was limited cross-program engagement amongst participants or peer mentors despite setting up the dedicated participant and peer mentor WhatsApp groups and encouraging the use of the “comments” function in Google Classroom. During the program feedback group discussion, two peer mentors noted that they enjoyed hearing about others’ experiences and wished they had connected sooner. One participant commented that she was nervous to share her work and ask questions in the participant group forum:

“One of the modules we had to write down our research questions [in the WhatsApp group]. I was very nervous (…) [about] the idea of having everybody reading and being like: ‘What was she thinking?’ (…) I think about three or so of us only posted our research questions, but I was very grateful because I was corrected (…) going forward, I think, as participants we do need to discuss and share ideas.” (PPH3)

Adaptations were made during program implementation. Mentorship teams were originally designed to include two participants, peer mentors, and faculty mentors each, however, only five peer mentors joined the program and thus the mentorship structure was modified to include just one peer mentor per team (***Figure 2***). The initial anticipation was that the program would be completed in four months, with a mix of one and two-week modules. However, after module three, received feedback showed that many participants were struggling with the pace of the program. Therefore, “catch-up” periods seen in ***Figure 1*** were added and all remaining modules were extended to two weeks, increasing the total program length to six months. It was initially a completion certificate requirement to participate in mentorship full team meetings. However, some teams experienced communication and scheduling difficulties since members came from numerous time-zones and some had inconsistent internet connectivity. Thus, this requirement was removed and teams were encouraged to meet in smaller groups or use email and/or WhatsApp for asynchronous communication as needed. Adaptability was highlighted as a strength of the pilot program: *The program was adaptable which helped a lot; (it) was important to give all the participants leeway*. (FRMH1)

Peer mentors reported investing an average of one to two hours per week in ARC activities. Two faculty mentors who were active in the program reported spending an average of one hour per week. Both felt this was appropriate, with FRMH1 noting faculty might “*run-away scared*” if asked to do more. Direct costs to provide an e-book to all participants and peer mentors totaled $450 USD. Out-of-pocket costs incurred by participants and mentors were not directly captured. However, network connectivity was a commonly cited program barrier and one individual reported purchasing a new modem to improve internet access. An eight-person working group, including two master of public health practicum students who dedicated over 250 hours each, provided the human resources needed to develop, implement, and evaluate the ARC program.

## DISCUSSION

### KEY FINDINGS

Results demonstrate that an online research training and mentorship program for early-career FPs in sub-Saharan Africa is both feasible and acceptable. Participants completed more than half of the total curriculum deliverables and four completed full research protocols during the six-month core program. Mentorship structures were established on all five teams with varying degrees and strategies of implementation. The most active mentors reported the time-commitment was still manageable and the overall financial cost of the pilot was low. Findings from the reach and adoption domains are promising for scalability. ARC reached participants across five African countries who were mostly postgraduate FP trainees. Participants joined primarily due to the desire to access research mentorship that is limited in their setting, which prior reports suggest is a widespread concern for FP trainees [8,9]. Adoption of this pilot by mentors from 11 countries across three continents demonstrates that FP researchers worldwide are willing and able to engage in distance mentorship to support trainees. These findings are especially relevant in the context of the covid-19 pandemic, which is pushing medical and postgraduate education online like never before [26,27].

### RESEARCH CULTURE DEVELOPMENT

One of the most promising successes of the pilot was the evidence of a developing primary health care research culture among active participants and the commitment to continue research activity and mentorship beyond the core program:

“Every day in clinical practice, all of a sudden I’m finding myself asking questions: What can I do to try and improve this? And for me it’s a sign that going forward, research will definitely be part of what I do.” (PPH3)

A program completion celebration was held to virtually present ARC completion certificates and acknowledge the hard work by all, responsive to suggestions for building research culture by Mash, et al [4]. A six-month ARC post-program period, comprised of monthly online research work-in-progress meetings is ongoing and participants and mentors who reach requirements over that period will also earn a certificate.

### RECRUITMENT CHALLENGES

It was difficult to recruit the intended number of peer mentors for an equal ratio of mentors and mentees; it was also a challenge to accurately assign mentors as a peer mentor or faculty mentor. One possible reason for low peer mentor adoption is the time-pressure experienced by early career FP researchers. A near-peer mentorship program in Uganda, in which masters students mentored undergraduate students, was limited by the near-peers’ heavy workload and conflicting priorities [28]. One peer mentor (anonymous) in our program noted: “*I didn’t feel competent in my mentorship skills during the first period of the program*.” A lack of confidence in mentoring others may have been another barrier to peer mentor recruitment. In addition, one of the mentors was mis-assigned as a peer mentor when she met the faculty mentor criteria by both training completion year and number of first-author publications. Also, two of the faculty mentors did not reach the first-author publication criteria. These oversights occurred because a method to systematically request and receive CVs from all mentors at the beginning of the program to cross-check self-report on application surveys was not in place. The resulting range of research experience among those assigned to the peer and faculty mentor role may have impacted team mentorship dynamics.

### MENTORSHIP STRUCTURE

The tiered mentorship team structure was designed to capitalize on peer-mentorships, which can flatten hierarchies [29] and extend limited faculty research capacity [10]. Peer relationships [30], and specifically peer-to-peer mentoring [31], have been cited as strategies to protect against burnout, which was an explicit goal of this program. This structure worked well when peer mentors were active in outreach to not only their peer mentees, but also their faculty mentors. However, if the peer mentor did not initiate and maintain the relationship with the faculty mentors, some faculty mentors ended up being left out of the team mentorship process entirely. On some teams this was partly due to confusion over responsibility for outreach: *“There should be an indication for who calls the meeting between the peer mentors and faculty mentors.”* (Anonymous PRM, Midway Evaluation).

Additionally, the participants were the target of the ARC program and thus the primary focus of peer mentor and participant relationship was clear: to check in each module and support the participant’s research protocol development. The primary focus of the peer mentor and faculty mentor relationship, however, was a broader goal of improving peer mentor’s mentorship skills. While peer mentors were encouraged at orientation to set learning goals and complete a mentorship reflection tool [32], it was not required. Mentorship contracts, which can clarify expectation and objectives [33], were also suggested but not required. Cole et al. highlight the importance of balancing structure and flexibility in global research mentorship programs [34]. The ARC pilot was lauded for its flexibility in terms of curriculum timeline, especially given participants and mentors alike experienced personal and professional challenges that impacted available time and energy. However, the mentorship portion required more structure and clarity, especially between peer mentors and faculty mentors:

*Suggestions for improvement in the future include (…) having more defined guidelines for team functioning, when to reach out, how much to reach out, how much faculty mentor feedback is desired or “enough*.” (FRMU2)

Participants and mentors largely limited mentorship interactions to their teams and did not utilize platforms to engage horizontally across the program. An evaluation of an online learning platform for postgraduate trainees in Germany reported that a perceived lack of involvement by other users can become a negative feedback loop [35]. Normalizing the utilization of cross-program structures for communication should be established at the beginning of the program. Creating opportunities for early and ongoing rapport-building both within and across teams may help participants and mentors feel these are safe spaces [36] where it is “*okay to demonstrate that you don’t know something*.” (FRMH1)

### FUTURE DIRECTIONS

The pilot study suggests a number of strategies that may improve upon reach, adoption, and implementation of online research training and mentorship programs for early career physicians in low-resource settings. Directly linking such programs to doctoral training or junior faculty development programs may not only provide increased opportunities for peer mentor recruitment, but also enhance the pipeline effect of these programs. The mentees are provided with an example to follow if they decide to pursue further training, and the early-career peer mentor gains skills in mentorship, which is equally crucial to building the pipeline [36]. The ARC pilot only provided mentorship training during orientation, however, results suggest that providing additional formal mentorship training sessions may have been of benefit. These results affirm calls for a greater focus on dedicated research mentorship training [7,37]. The curriculum itself was generally well received, however some participants requested more focus on research methodology. Programs such as ours should consider providing funds to offset costs to participants with limited internet access and offering training materials and mentors proficient in languages other than English to avoid exacerbating existing inequities in access to research training opportunities [38].

### LIMITATIONS

The small size of this pilot, which was restricted by available resources, limits interpretation of our findings. Our recruitment methods did not allow for analysis of non-participants and non-adopters, a key component of the reach and adoption domains [17]. Our mentorship team evaluation relied largely on reports by the peer mentors, and may have missed faculty mentor mentorship activity as a result. Additionally, since the authors were involved in development, implementation, and evaluation of the program, the findings may be subject to both researcher bias and response bias from participants and mentors. Using mixed-methods and multiple sources of data, some of which were anonymous, to triangulate our findings helps to mitigate these limitations. Furthermore, authors’ use of reflexive memoing, regular debriefing, and the use of member checking of results help to ensure the validity of our findings.

## CONCLUSIONS

It is both feasible and acceptable to conduct online research training and mentorship for trainee and early career FPs in sub-Saharan Africa. Similar programs must pay careful attention to mentorship training and provide a flexible yet clearly organized structure for mentee-mentor engagement. Future work is needed to determine optimal implementation strategies and to scale up efforts to develop primary health care research capacity in sub-Saharan Africa.

## Figures and Tables

**Supporting Figure S1:**
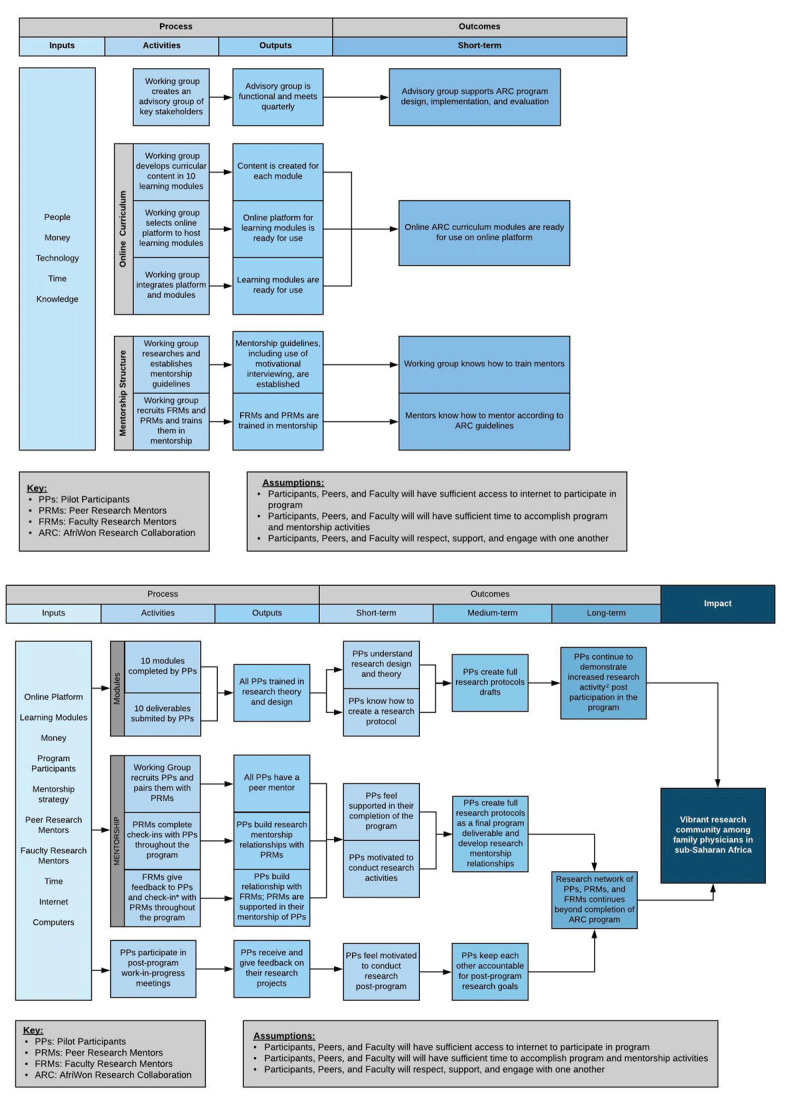
ARC Pilot Target and non-Target Logic Models used to Design Program and Evaluation. Abbreviations: See Figure Keys. x Non-target refers to programmatic inputs, including ARC mentors. y Target refers to ARC pilot participants. z Research activities include engaging in research projects, participating in research discussions with colleagues, providing or receiving research mentorship; presenting and disseminating research outputs.

## APPENDIX

**Supporting Table S2:** ARC Pilot Program Process Evaluation Data Collection and Management.

DATA TYPE	VARIABLES COLLECTED/TIMING	SOURCE/SAMPLING	MONITORING AND EVALUATION MANAGEMENT AND UTILIZATION

Recruitment Applications and Log, Baseline PP Data	**Variables:** Demographic and research activity data; PP self-perceived level of burnout (using a single-item validated burnout measure) [39]; recruitment numbers **Timing:** Once at beginning of program, apart from PRM CVs collected at program end	**Sources:** Electronic applications, PRM and FRM CVs, Baseline PP Survey **Sampling:** All PPs, PRMs, and FRMs	Recruitment data reviewed at each Working Group meeting throughout ARC recruitment and onboarding period; Demographic characteristics of PPs aggregated and presented at Advisory Group quarterly meeting (after module 3)

Deliverable Log	**Variables:** Deliverable completion status by pilot participant (PP); timeliness of submission **Timing:** Each module end	**Source:** Google Classroom/ARC Email account **Sampling:** All PPs	Email regarding deliverable sent to PP and all mentors at each module end; Aggregate data presented to working group after modules 3, 6, and 10

Mentorship Log	**Variables:** (1) number of “check-ins” between each team member, (2) platform used for communication, (3) duration, (4) content covered, (5) any challenges expressed by the PP, and (6) other mentorship observations **Timing:** Each module end	**Source:** Working Group liaison WhatsApp discussion with peer research mentor **Sampling:** All PRMs (who reported on all members of team)	Informal presentation by Working Group liaison every two weeks at working group meeting; Aggregate data reviewed after modules 3 and 10; Aggregate review conducted by a second working group member and overall findings written as two (early, #1, and late, #2) ARC mentorship summary documents

Midway and Final Program Evaluations	**Variables:** Perception of 1) curriculum, 2) mentorship (adapted from validated questionnaire),[19] and 3) ARC program delivery **Timing:** Midway survey sent after module 6; Final survey sent after module 10	**Source:** Online anonymous program evaluation surveys **Sampling:** All PPs, PRMs, FRMs	Aggregate midway mentorship results sent to PRMs and FRMs; Summary of all evaluation sent to module lecturers; Survey data reviewed at regular Working Group meetings and used to guide development of questions for feedback group discussions (below)

Program Feedback Group Discussion	**Variables:** Experience during program, including average time per week spent on ARC activities, and ideas for future of ARC **Timing:** After module 8	**Source:** Meeting minutes including key quotes from video recording **Sampling:** All PPs, PRMs, FRMs invited	Findings discussed during regular Working Group meetings; feedback used to guide post-program activities

Program Logs/Expense log	**Variables:** Observations on PP and mentor use of WhatsApp and Google Classroom platforms; record of major pilot program activities and adaptations made; Program costs, such as e-books and transcription software **Timing:** Rolling throughout	**Sources:** Working Group recorded observations, Email receipts **Sampling:** All teams monitored; all major program activities captured; all costs noted	An early (after module 3) and late (after module 10) summary of observations from program logs was created; both were reviewed at regular Working Group meetings; Expenses discussed regularly during Working Group meetings; Midway report shared with funder (Consortium of Universities for Global Health)

Abbreviations: ARC, AfriWon Research Collaborative; PP, Pilot Participant; PRM, Peer Research Mentor; FRM, Faculty Research Mentor; CV, Curriculum Vitae.

## References

[1] Starfield B, Shi L, Macinko J. Contribution of primary care to health systems and health. Milbank Q. 2005; 83(3): 457–502. DOI: 10.1111/j.1468-0009.2005.00409.x 16202000 PMC2690145

[2] Ponka D, Coffman M, Fraser-Barclay KE, et al. Fostering global primary care research: A capacity-building approach. BMJ Glob Health. 2020; 5(7): e002470 DOI: 10.1136/bmjgh-2020-002470 PMC7337619 32624501

[3] Mar CD, Askew D. Building family/general practice research capacity. Ann Fam Med. 2004; 2(suppl 2): S35–S40. DOI: 10.1370/afm.146 15655086 PMC1466770

[4] Mash R, Essuman A, Ratansi R, et al. African primary care research: Current situation, priorities and capacity building. Afr J Prim Health Care Fam Med. 2014; 6(1). DOI: 10.4102/phcfm.v6i1.758 PMC5326807 26245447

[5] van Weel C. The impact of research in primary care and family medicine: The Thomson Reuters Web of Science Subject Category “Primary Health Care.” Fam Pract. 2011; 28(3): 239–240. DOI: 10.1093/fampra/cmr021 21602287

[6] Flinkenflögel M, Essuman A, Chege P, Ayankogbe O, De Maeseneer J. Family medicine training in sub-Saharan Africa: South–south cooperation in the Primafamed project as strategy for development. Fam Pract. 2014; 31(4): 427–436. DOI: 10.1093/fampra/cmu014 24857843 PMC4106404

[7] McGuire CM, Yakubu K, Ayisi-Boateng NK, et al. Exploring gaps, strategies and solutions for primary care research mentorship in the African context: A workshop report. Afr J Prim Health Care Fam Med. 2020; 12(1): 4 DOI: 10.4102/phcfm.v12i1.2320 PMC7284154 32501030

[8] Dubale BW, Friedman LE, Chemali Z, et al. Systematic review of burnout among healthcare providers in sub-Saharan Africa. BMC Public Health. 2019; 19(1): 1–20. DOI: 10.1186/s12889-019-7566-7 31510975 PMC6737653

[9] Yakubu K, Colon-Gonzalez MC, Hoedebecke K, Gkarmiri V, Hegazy NN, Popoola OO. Meeting report: ‘How do I incorporate research into my family practice?’: Reflections on experiences of and solutions for young family doctors. Afr J Prim Health Care Fam Med. 2018; 10(1): 6 DOI: 10.4102/phcfm.v10i1.1640 PMC5913785 29781695

[10] Phipps W, Kansiime R, Stevenson P, Orem J, Casper C, Morrow RA. Peer mentoring at the Uganda Cancer Institute: A novel model for career development of clinician-scientists in resource-limited settings. J Glob Oncol. 2018; 4 DOI: 10.1200/JGO.17.00134 PMC6223430 30241258

[11] Lucas H, Kinsman J. Distance- and blended-learning in global health research: Potentials and challenges. Glob Health Action. 2016; 9 DOI: 10.3402/gha.v9.33429 PMC5056981 27725082

[12] Shrestha CH, May S, Edirisingha P. From face-to-face to e-mentoring: Does the “e” add any value for mentors? Int J Teach Learn High Educ. 2009; 20(2): 116–124.

[13] Global Family Doctor – WONCA Online: Young Doctors’ Movements. Accessed June 17, 2020. https://www.globalfamilydoctor.com/groups/YoungDoctorsMovements.aspx

[14] Yakubu K, Hoedebecke K, Nashat N. Young doctor movements: Motives for membership among aspiring and young family physicians. J Fam Med Prim Care. 2015; 4(2): 177–181. DOI: 10.4103/2249-4863.154625 PMC4408696 25949962

[15] Goodyear-Smith F, Mash R. How to Do Primary Care Research 1st ed. CRC Press; 2018 DOI: 10.1201/9781351014519

[16] Miller WR, Rose GS. Toward a theory of motivational interviewing. Am Psychol. 2009; 64(6): 527–537. DOI: 10.1037/a0016830 19739882 PMC2759607

[17] Glasgow RE, Harden SM, Gaglio B, et al. RE-AIM Planning and Evaluation Framework: Adapting to new science and practice with a 20-year review. Front Public Health. 2019; 7 DOI: 10.3389/fpubh.2019.00064 30984733 PMC6450067

[18] R Core Team. R: A Language and Environment for Statistical Computing R Foundation for Statistical Computing; 2019 https://www.R-project.org/

[19] NVivo Qualitative Data Analysis Software. QSR International Pty Ltd; 2018.

[20] Braun V, Clarke V. Using thematic analysis in psychology. Qual Res Psychol. 2006; 3(2): 77–101. DOI: 10.1191/1478088706qp063oa

[21] Birks M, Chapman Y, Francis K. Memoing in qualitative research: Probing data and processes. J Res Nurs. 2008; 13(1): 68–75. DOI: 10.1177/1744987107081254 PMC12602296 41229605

[22] Kleinsasser AM. Researchers, reflexivity, and good data: Writing to unlearn. Theory Pract. 2000; 39(3): 155–162. DOI: 10.1207/s15430421tip3903_6

[23] Bamberger M, Rugh J, Mabry L. Real World Evaluation: Working Under Budget, Time, Data, and Political Constraints Second Edition. SAGE Publications, Inc; 2012.

[24] Creswell JW, Miller DL. Determining validity in qualitative inquiry. Theory Pract. 2000; 39(3): 124–130. DOI: 10.1207/s15430421tip3903_2

[25] Global Family Doctor – WONCA Online. Accessed April 14, 2020. https://www.globalfamilydoctor.com/AboutWonca/Regions.aspx

[26] Kanmounye US, Esene IN. Letter to the editor “COVID-19 and neurosurgical education in Africa: Making lemonade from lemons.” World Neurosurg. Published online 5 21, 2020 DOI: 10.1016/j.wneu.2020.05.126 PMC7241362 32446982

[27] Almarzooq Z, Lopes M, Kochar A. Virtual learning during the COVID-19 pandemic: A disruptive technology in graduate medical education. J Am Coll Cardiol. Published online 4 15, 2020 DOI: 10.1016/j.jacc.2020.04.015 PMC7159871 32304797

[28] Rukundo GZ, Burani A, Kasozi J, et al. Near-peer mentorship for undergraduate training in Ugandan medical schools: Views of undergraduate students. Pan Afr Med J. 2016; 23 DOI: 10.11604/pamj.2016.23.200.7691 PMC4907755 27347289

[29] Prasad S, Sopdie E, Meya D, Kalbarczyk A, Garcia PJ. Conceptual framework of mentoring in low- and middle-income countries to advance global health. Am J Trop Med Hyg. 2019; 100(1 Suppl): 9–14. DOI: 10.4269/ajtmh.18-0557 PMC6329351 30430983

[30] Dyrbye L, Shanafelt T. A narrative review on burnout experienced by medical students and residents. Med Educ. 2016; 50(1): 132–149. DOI: 10.1111/medu.12927 26695473

[31] Ironside K, Becker D, Chen I, et al. Resident and faculty perspectives on prevention of resident burnout: A focus group study. Perm J. 2019; 23 DOI: 10.7812/TPP/18-185 PMC6636525 31314728

[32] Fleming M, House S, Shewakramani V, et al. The Mentoring Competency Assessment: validation of a new instrument to evaluate skills of research mentors. Acad Med J Assoc Am Med Coll. 2013; 88(7): 1002–1008. DOI: 10.1097/ACM.0b013e318295e298 PMC3727250 23702534

[33] Cooke KJ, Patt DA, Prabhu RS. The road of mentorship. Am Soc Clin Oncol Educ Book. 2017; (37): 788–792. DOI: 10.1200/EDBK_175193 28561670

[34] Cole DC, Johnson N, Mejia R, et al. Mentoring health researchers globally: Diverse experiences, programmes, challenges and responses. Glob Public Health. 2016; 11(9): 1093–1108. DOI: 10.1080/17441692.2015.1057091 26234691 PMC5020346

[35] Dini L, Galanski C, Döpfmer S, et al. Online platform as a tool to support postgraduate training in general practice – A case report. GMS J Med Educ. 2017; 34(5). DOI: 10.3205/zma001136 PMC5704610 29226227

[36] Bartels SJ, Lebowitz BD, Reynolds CF, et al. Programs for developing the pipeline of early-career geriatric mental health researchers: Outcomes and implications for other fields. Acad Med J Assoc Am Med Coll. 2010; 85(1): 26–35. DOI: 10.1097/ACM.0b013e3181c482cb PMC2931586 20042817

[37] Katz F, Glass RI. Mentorship training is essential to advancing global health research. Am J Trop Med Hyg. 2019; 100(1 Suppl): 1–2. DOI: 10.4269/ajtmh.18-0694 PMC6329355 30430975

[38] Minja H, Nsanzabana C, Maure C, et al. Impact of health research capacity strengthening in low- and middle-income countries: The case of WHO/TDR programmes. PLoS Negl Trop Dis. 2011; 5(10): e1351 DOI: 10.1371/journal.pntd.0001351 22022630 PMC3191138

[39] Dolan ED, Mohr D, Lempa M, et al. Using a single item to measure burnout in primary care staff: A psychometric evaluation. J Gen Intern Med. 2015; 30(5): 582–587. DOI: 10.1007/s11606-014-3112-6 25451989 PMC4395610

